# The scent of fear makes sea urchins go ballistic

**DOI:** 10.1186/s40462-021-00287-1

**Published:** 2021-10-09

**Authors:** Jordi F. Pagès, Frederic Bartumeus, Javier Romero, Teresa Alcoverro

**Affiliations:** 1 Departament de Biologia Evolutiva, Ecologia i Ciències Ambientals, Facultat de Biologia, Universitat de Barcelona, Barcelona, Spain; 2 Centre d’Estudis Avançats de Blanes (CEAB-CSIC), Blanes, Spain; 3 Institució Catalana de Recerca i Estudis Avançats (ICREA), Barcelona, Spain; 4 Centre de Recerca Ecològica i Aplicacions Forestals (CREAF), Cerdanyola del Vallès, Spain; 5 Nature Conservation Foundation, Amritha, 1311, 12th Cross, Vijayanagar 1st Stage, Mysore, 570017 India

**Keywords:** Animal movement, Chemical cue, Escape response, Fear, Predator–prey

## Abstract

**Background:**

Classic ecological formulations of predator–prey interactions often assume that predators and prey interact randomly in an information-limited environment. In the field, however, most prey can accurately assess predation risk by sensing predator chemical cues, which typically trigger some form of escape response to reduce the probability of capture. Here, we explore under laboratory-controlled conditions the long-term (minutes to hours) escaping response of the sea urchin *Paracentrotus lividus*, a key species in Mediterranean subtidal macrophyte communities.

**Methods:**

Behavioural experiments involved exposing a random sample of *P. lividus* to either one of two treatments: (i) control water (filtered seawater) or (ii) predator-conditioned water (with cues from the main *P. lividus* benthic predator—the gastropod *Hexaplex trunculus*). We analysed individual sea urchin trajectories, computed their heading angles, speed, path straightness, diffusive properties, and directional entropy (as a measure of path unpredictability). To account for the full picture of escaping strategies, we followed not only the first instants post-predator exposure, but also the entire escape trajectory. We then used linear models to compare the observed results from control and predators treatments.

**Results:**

The trajectories from sea urchins subjected to predator cues were, on average, straighter and faster than those coming from controls, which translated into differences in the diffusive properties and unpredictability of their movement patterns. Sea urchins in control trials showed complex diffusive properties in an information-limited environment, with highly variable trajectories, ranging from Brownian motion to superdiffusion, and even marginal ballistic motion. In predator cue treatments, variability reduced, and trajectories became more homogeneous and predictable at the edge of ballistic motion.

**Conclusions:**

Despite their old evolutionary origin, lack of cephalization, and homogenous external appearance, the trajectories that sea urchins displayed in information-limited environments were complex and ranged widely between individuals. Such variable behavioural repertoire appeared to be intrinsic to the species and emerged when the animals were left unconstrained. Our results highlight that fear from predators can be an important driver of sea urchin movement patterns. All in all, the observation of anomalous diffusion, highly variable trajectories and the behavioural shift induced by predator cues, further highlight that the functional forms currently used in classical predator–prey models are far from realistic.

**Supplementary Information:**

The online version contains supplementary material available at 10.1186/s40462-021-00287-1.

## Background

Prey organisms have evolved a variety of antipredator strategies to enhance their probability of survival in the face of predation. These adaptations may reduce the probability of actually encountering a predator, or the probability of consumption once the prey has been detected [1]. Most prey are capable of accurately assessing predation risk [2], some of them using multiple predator detection mechanisms—including visual, chemical and/or tactile cues. However, chemical detection appears to be the most reliable way to perceive the presence of current and past predators [3], in the latter case providing advanced warning of danger [4], and thus allowing the possibility of avoiding the risk of being preyed upon altogether. Predator chemical detection occurs in terrestrial, freshwater and marine organisms [3]. Once the cue has been detected, a behavioural reaction is usually triggered. In the case of early predator detection, avoidance (e.g. refuge seeking) is among the most common responses, but when the attack is inevitable or already initiated, the most common strategy is to reduce the probability of capture by escaping [5]. Since antipredator behaviour, however, incurs in a cost in the form of lower access to resources and/or lower metabolic rate, it would be a selective advantage to assess the need to escape or shelter in an accurate manner to optimally balance potential risks (predation) with potential gains (food intake) [6]. Antipredator behaviour, therefore, is a typical example of how an animal needs to rapidly integrate information from its environment to produce an appropriate behavioural response that is constrained by the animal’s body condition, biomechanics, and information processing capabilities [5].

Classic ecological formulations of predator–prey interactions (e.g. Lotka–Volterra equations) often assume that predators and prey interact randomly, in a manner similar to molecules in an ideal gas undergoing Brownian motion [7, 8], and therefore the probability of encounter depends mostly on their respective concentrations (densities). In this idealised scenario, neither of them receives external information about each other’s position, and predation only occurs when the movement trajectories of both predator and prey coincide. Although a lot of research has focused on generalising how predators find and process prey once found (following [9]), the inclusion of individual-level movement patterns in population models is still not common. One of the few examples where the differences in mobility and speed have been incorporated in predator–prey models showed that predator foraging mode (mobile vs sit-and-wait) controlled the success of prey antipredator behaviour, which influenced community stability [10]. Also, in a recent paper, Hein and Martin [11] found that accounting for the ‘information limitation’ that a predator experiences when looking for prey can stabilize predator–prey systems, preventing the collapse of predator and prey populations. However, both papers mainly focus on predator movements, while still assuming that prey move at random. To the best of our knowledge, prey escaping strategies have rarely been incorporated in predator–prey models [10, 12, 13].

To date, a lot of research has focused on the initial stages of prey escape using instantaneous measurements, such as the escape direction, speed or acceleration [5, 14]. Theoretical models show that on the basis of the relative speeds of predator and prey, a single optimal escape trajectory can be predicted [14, 15]. However, these predictions on prey responses to predator strikes, do not accommodate one of the main properties postulated for escape trajectories—their unpredictability, which seems fundamental for preventing predators from learning a repeated pattern of prey response [16]. Often, escape responses not only involve a rapid stereotyped retreat phase (rapid relative to the predator’s movement), but also some degree of so-called ‘protean behaviour’ with unpredictable escape patterns [1]. While it is true that in many taxa, the first milliseconds after predator attack are crucial for predator escape [17], this is not always the case. For example, when escaping from pursuit predators (those that do not ambush and strike their prey), the complexity of the escape response during an extended period of time can be a key element for understanding the factors that lead to surviving predation [5]. Thus, to account for the full picture of escaping strategies, we need to follow not only the first vital milliseconds post-predator attack, but also the entire escape trajectory. In addition, to bring together behaviour with population dynamics, we need to characterise not only predator search behaviour (as successfully done by Hein & Martin [11]), but we also need to understand that prey escaping trajectories might deviate from the ideal gas models of animal encounter [8, 18].

Sea urchins (class *Echinoidea*) are a group of marine invertebrates with an old evolutionary origin [19, 20] and lack of cephalization [21] that play a very important functional role in marine benthic ecosystems (e.g. some behaving as key herbivores [22]). They display several adaptations for sensing their environment and for detecting potential predators. Some species display behavioural responses to dark objects and/or light [23–25], while others also display chemosensory abilities to detect predators [26–30], or detect cues from damaged conspecifics [29–32]. However, the study of the responses to these stimuli in terms of movement patterns is generally limited to the comparison of the heading angles before-after detection and distance travelled (e.g. [29, 31]); whereas their response to predators, in terms of changes in their trajectories and diffusive properties, has not been studied, to the best of our knowledge.

Here, we explore under laboratory-controlled conditions the long-term (minutes to hours) heading angles, trajectories, diffusive properties, and predictability of movement of the sea urchin *Paracentrotus lividus* (Lam.). *P. lividus* is widely recognised as a key species in Mediterranean subtidal macrophyte communities, given its role as one of the main grazers in rocky reefs and *Posidonia oceanica* seagrass meadows [33–35]. We assess the role of predator chemical cues in conditioning average sea urchin movement behaviour and discuss the consequences of the emerging diffusive properties on predator–prey encounter rates and on potential prey survival. Behavioural experiments involved exposing a sample of *P. lividus* sea urchins to either one of two treatments: (i) control water (filtered seawater) and (ii) predator-conditioned water, that held overnight 6 individuals of the main *P. lividus* benthic predator—the slow-moving gastropod *Hexaplex trunculus* (L.). Given that the chemical stimulus used in this experiment was diffuse, we expect uniformly distributed heading angles both for control and predator treatments. If sea urchins perceive predator cues, we also expect straighter or faster trajectories at the predator trials. Finally, it is not clear whether sea urchin escape responses to predator cues will turn out to be predictable, if the priority is to depart from the predator, or unpredictable, if the intention is to mislead the predator, avoiding it from learning the optimal escape trajectory [1].

## Methods

### Specimen collection and care

On the day before the start of each round of trials, we collected *Paracentrotus lividus* sea urchins (test diameter without spines 3.6 ± 0.05 cm, mean ± SE) and predatory gastropods *Hexaplex trunculus* (ca. 8 cm in length), from a shallow (2–4 m) macroalgal-dominated habitat in Blanes (41° 40' N 2° 48' E; 41° 44' N 2° 57' E). We kept all sea urchins and gastropods in aquaria with the same light cycle and similar temperature as those in the field and with a continuous flow of seawater (mean salinity 35.8 ± 0.2 psu, mean temperature 23.1 ± 0.5°C). All urchins and gastropods were fed ad libitum (macroalgae and live sea urchins respectively, although gastropods did not consume any of the sea urchins offered) and were tested within a week of collection. We ran some initial trials with animals differing in acclimation duration. We observed that animal speeds and displacements from the initial point decreased in animals that had been kept in the holding aquaria for more than a week (likely a caging effect). As a result, we decided to run the trials between 2- and 5-days post-capture, thus, after a minimum of one day of acclimation in the holding aquaria. Such first trials allowed us to confirm that the responses that sea urchins displayed while in the experimental arena were similar to those shown when displaced in the field: they typically move away from the initial point if they do not find a refuge right away; otherwise, when they find a refuge, they might not move for hours. Each sea urchin was randomly allocated to one of two treatments, namely: control—with filtered seawater; and predator treatment—with filtered seawater that had been conditioned with chemicals from the predatory gastropod *H. trunculus*. We routinely tested the capacity of sea urchins to reverse when put upside-down (with animals that would not be tested on that day), to check their health status. We considered the animals were not in good condition when they were not capable of overturning themselves in less than 5 min, in which case they were discarded.

### Experimental arena

**Fig. 1:**
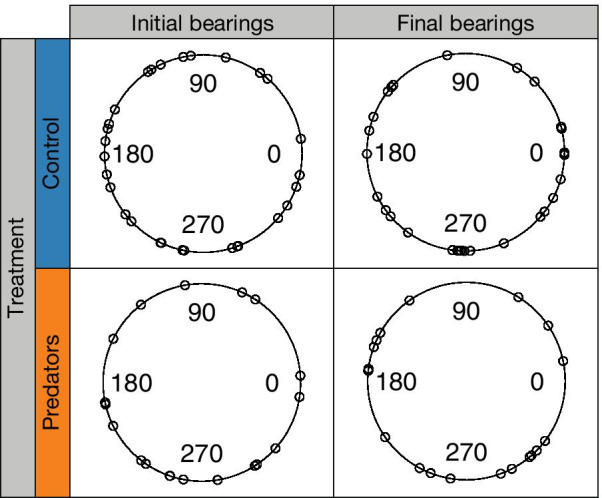
Sea urchin final and initial distribution of heading angles in the control (n = 29) and predator (n = 21) treatments. The distribution of angles was considered uniform according to Rayleigh tests both for initial control (z = 0.16, *P*-value = 0.46), initial predator (z = 0.23, *P*-value = 0.32), final control (z = 0.21, *P*-value = 0.29) and final predator trials (z = 0.19, *P*-value = 0.48)

The experimental arena consisted of a metallic circular tank (3 m diameter) lined with plastic. A diffuse fluorescent light source was placed 3 m above the arena resulting in a mean downwelling irradiance on the arena’s floor of 7.7 × 10^18^ photons m^−2^ s^−1^ (measured with a HOBO, Amplified Quantum Sensor, model SQ-200, Onset Computer Corporation, USA). Despite attempts to ensure the arena was homogenously lit, we found some heterogeneity in downwelling irradiance (Additional file 1: Fig. S1). However, such heterogeneity was not enough to bias sea urchin directionality (see Fig. 1). Three metres above the arena, we mounted a Nikon D80 (Nikon, Japan) digital SLR camera with a 17 mm lens that allowed a full view of the experimental area. We set the camera in time-lapse mode with a 30-s interval between each image, since preliminary testing had shown that this time interval allowed sea urchins to move approximately one body length between photographs. This temporal resolution is lower than that of other works using the same model species [36], but similar to studies interested in sea urchin movement behaviour over longer periods of time (e.g. [37, 38]). In fact, due to the small velocities of sea urchins, finer scale resolution might lead to results being largely affected by recording error.

### Experimental procedure

On each day of trials, the arena was filled (water column = 20 cm) either with filtered seawater at the same temperature and salinity as that of the holding aquaria (control seawater), or with seawater at the same temperature and salinity conditions but conditioned with chemical cues from the predatory gastropod *Hexaplex trunculus* (L), which is considered the main benthic predator of *P. lividus* sea urchins in the north-western Mediterranean [39]. Predator-conditioned seawater was obtained by allowing six individuals of the predatory gastropod to roam overnight in a tank containing the water that would be used for the experiment the following morning. Control seawater or predator-conditioned seawater was changed (the arena was refilled) on each day of trials, and at the end of each day of trials the arena was thoroughly scrubbed with a brush and clean seawater. Each sea urchin was tested only once. At the beginning of each trial a sea urchin was placed at the centre of the arena, and we considered the trial had ended when a sea urchin had approached to within 10 cm of the edge. While it would have been more realistic to slowly add control or predator-conditioned water to tanks with plain seawater containing urchins that had been allowed to settle down beforehand, we chose feasibility and experimental control, at the expense of similarity to field conditions. After the trial, the arena was scrubbed to reduce the potential for trail following and the sea urchin was returned to the holding aquaria and never used again. Twenty-nine sea urchins were used in control seawater and 21 for the predator treatment. Such difference in the replication level for each group was due to the availability of sea urchins, technical problems while running experiments (e.g. camera not taking the photos at the desired rate, unexpected visits to the lab while the experiment was running, etc.) and time constraints.

### Image and trajectory analysis

A total of 3337 images were taken. These were transferred to a computer and the x and y coordinates of each urchin were obtained by means of a Matlab script using the image processing toolbox. These coordinates were then transferred to R [40] and analysed with the package adehabitatLT [41], which computed step displacements in X and Y coordinates of the trajectory (frame rate = 30 s). We calculated the initial angle headings for each sea urchin with the arctangent of the vector between the first and fifth steps, while the final angle headings were calculated using the vector connecting the first and last steps (see diagram in Additional file 2: Fig. S2). We used the fifth step to calculate the initial headings, because we considered that by then, sea urchins would have had enough time (> 2 min) to reorient themselves after being moved from the holding tank to the experimental arena.

For each sea urchin, we also calculated its mean speed—by estimating speed for every step, and then averaging the values for the entire trajectory—and a measure of path tortuosity, the straightness index. The straightness index (I_s_) is a dimensionless number that ranges from 1 (maximum straightness) to 0 (maximum tortuosity), which is computed as the ratio of the Euclidian distance between the initial and final point of the trajectory and the sum of Euclidian distances between pairs of points separated by a given time (i.e., window width). Since different windows of time result in different I_s_ [42], we calculated this index for a range of window widths. Comparisons between control and predator treatment were consistent regardless of window width and, thus, here we only present I_s_ calculated using a window of 1 step (30 s).

We also analysed the spreading (diffusive) properties of sea urchin trajectories in each treatment. We used a general numerical approach estimating the q^th^ order long-range correlations of the sea urchin displacements [43]. Specifically, the module of increments of two-dimensional displacements is ∥∆Xτ∥ ≡ √(x_t+τ_ − x_t_)^2^ + (y_t+τ_ − y_t_)^2^, where τ is the temporal increment, and (x_t_, y_t_) and (x_t+τ_,y_t+τ_) are respectively the positions of a sea urchin at time t and t + τ. The moments of order q (q > 0) of the module of two-dimensional displacements depend on the temporal increment τ as

1 $$\left\langle {\parallel \Delta {\text{X}}\tau \parallel^{{\text{q}}} } \right\rangle \sim \tau^{{\zeta ({\text{q}})}}$$

The exponents ζ(q) were estimated as the slope of the linear regressions of ∥∆Xτ∥ vs. τ in log–log plots [43] (see Additional file 3: Fig. S3 for two examples of such estimation). To avoid autocorrelation and hence violation of independence (the most important assumption of linear regression, Zuur et al. 2009), we calculated ∥∆Xτ∥ using log-spaced temporal increments (τ), to obtain equally spaced observations in log–log plots, as recommended in [44]. The moment function ζ(q) characterises the statistics of the random walk ∥∆Xτ∥ of *P. lividus* regardless of the scale and intensity [45], and the related diffusive properties. Low and high orders of moment q characterise small, frequent displacements, and large, less frequent displacements, respectively. The mean (q = 1) and the variance (q = 2) are not sufficient to quantify the behaviour of probability density functions. A complete description requires an infinite number of moments (of q’s), hence the use of the whole function ζ(q) instead of a single exponent [43]. Each individual-level function ζ(q) was plotted along with the results for the Brownian motion (dashed line in Fig. 3b,d) and ballistic motion (dotted line in Fig. 3b,d). With this analysis we quantified the nature of the diffusive properties of sea urchin trajectories and discern whether these were subdiffusive (extremely restricted movement patterns), Brownian (local movements represented by short displacement lengths and tortuous trajectories), superdiffusive (including a large range of displacement lengths, accounting for both local and extensive movements), or ballistic (straight-line motion). Unfortunately, sea urchin trajectories were not long enough to analyse step length distributions, which can be used to discern the mechanisms underlying the diffusive anomalies observed.

We finally calculated the entropy of individual sea urchin trajectories as a measure of unpredictability of urchin movement patterns. It has been shown that unpredictability of animal trajectories can increase when escaping from predators to prevent them from learning a stereotyped evasion strategy (e.g. [46]). However, such strategy might be dependent on predator foraging mode. To calculate entropy for each individual sea urchin trajectory, we first discretised the relative angles between steps by binning them using the function cut() in R. After several trials, and seeing that the results did not change overall, we selected a bin width of 0.05 radians. We then calculated entropy as the Shannon H index of the resulting vector of relative angle bin counts (R package ‘entropy’) [47].

### Data analysis

Given that the predator chemical cue was diffuse in this experiment, we expected uniformly distributed heading angles (initial and final) both for control and predator treatments. A non-uniform distribution of the angles would be indicative of experimental artefacts (e.g., urchins orienting in response to room features, light heterogeneity, etc.). Thus, we used the Rayleigh test on the absolute initial and final heading angles for control and treatment sea urchins to ensure the animals were not orienting to stimuli other than the predator cues. The null hypothesis of the Rayleigh test is a uniform distribution of the heading angles, and the alternative hypothesis is a unimodal distribution with unknown direction (but with directionality). Rayleigh tests indicated that *Paracentrotus lividus* sea urchins displayed a uniform distribution of both the initial and final heading angles, both for the controls and the treatment with predator chemical cues (Fig. 1). Thus, sea urchins did not orient towards any unexpected feature of the arena or the room for any of the treatments (Fig. 1). The required statistical assumptions (i.e., unimodality and von-Misses distribution) were tested and fulfilled in all cases.

To assess if sea urchins increased the speed and straightness of their movement patterns when a predator cue was detected, we analysed the response variables ‘mean sea urchin speed’ and ‘straightness index’ with linear models using the fixed factor ‘treatment’ (2 levels: control, predator). We also checked whether the random effect ‘day of trial’ was needed to account for the shared variability among those individuals tested on the same day. However, this random effect did not improve the model according to the Akaike Information Criterion [48], and was therefore not included in the final models. Normality and homoscedasticity were assessed (visual inspection of residuals) and fulfilled for both mean sea urchin speeds and straightness index.

To assess if sea urchin diffusive properties differed in presence of predator chemical cues, we used a Generalised Least Squares (GLS) model with the response variable ‘scaling exponents of the function ζ(q)’ as a function of the fixed factor ‘treatment’ (2 levels: control, predator). We used a GLS instead of a plain linear model because we observed a clear violation of homoscedasticity assumption between treatments. Data exploration revealed a much greater variance in the diffusive properties of the control group than that of the predator treatment group (Levene’s Test *p *value = 0.001). To account for such treatment-level heterogeneity, we included the factor ‘treatment’ as weights in the GLS model, using the function varIdent() from the package nlme in R [48, 49]. This function allows the variance to vary between predictor levels, i.e., between control and predator treatments in our case. Again, we tested the need for including the random effect ‘day of trial’ for this response variable, but the Akaike Information Criterion did not support its inclusion in the final model (there was no shared variance among the individuals tested on the same day) [48]. Normality and homoscedasticity were assessed (visual inspection of residuals) and fulfilled after including the treatment-level variance structure as weights.

Finally, we assessed whether the predictability of trajectories differed between sea urchins moving in a featureless arena and sea urchins exposed to predator chemical cues. To this end, we fitted a linear model with the response variable ‘entropy of the distribution of relative angles’ (continuous) and ‘treatment’ as a fixed factor (2 levels: control, predator), plus the continuous fixed variable ‘scaling exponents of the function ζ(q)’, to check if there was a relationship between the predictability of sea urchin trajectories (directional entropy) and sea urchin diffusive properties (scaling exponents of the function ζ(q)). We also checked whether the random effect ‘day of trial’ was needed to account for the shared variability among those individuals tested on the same day. However, this random effect did not improve the model according to the Akaike Information Criterion [48], and was therefore not included in final models. Normality and homoscedasticity were assessed (visual inspection of residuals) and fulfilled.

Each individual sea urchin was always considered a replicate, in all analyses. Despite the number of replicates between control and treatment groups differed, current implementations of linear and generalised least squares models can perfectly solve the equations for unbalanced designs [50]. All analyses were performed in R v3.6.0 [40], and the R scripts used to run all of the analyses reported are available as a GitHub repository (available here [51]).

## Results

**Fig. 2:**
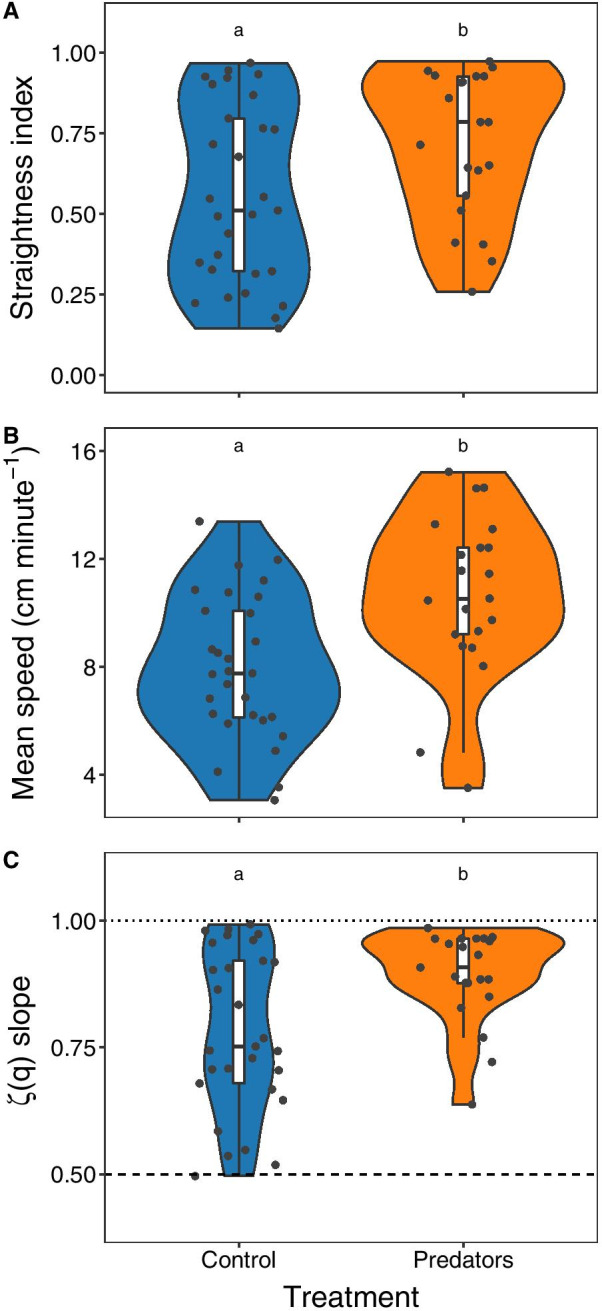
Violin plots of the different sea urchin movement variables analysed: **a** Sea urchin trajectories were on average straighter (less tortuous) and **b** faster (higher mean speeds) in the predator treatment (n = 21) compared to controls (n = 29). **c** Scaling exponents of the qth order structure functions indicated that, on average, sea urchins shifted from a wide range of spreading patterns, including Brownian motion, in control conditions to superdiffusive and marginal ballistic motion in the presence of predator chemical cues. The dashed and dotted lines in panel **c** represent the theoretical slope of purely Brownian and ballistic motion respectively. Different lower-case letters indicate statistically significant differences (see also Table 1). Shaded areas (‘violin-plots’) illustrate the kernel probability density of the data for each experimental treatment. Black points correspond to each individual observation (each sea urchin)

**Fig. 3:**
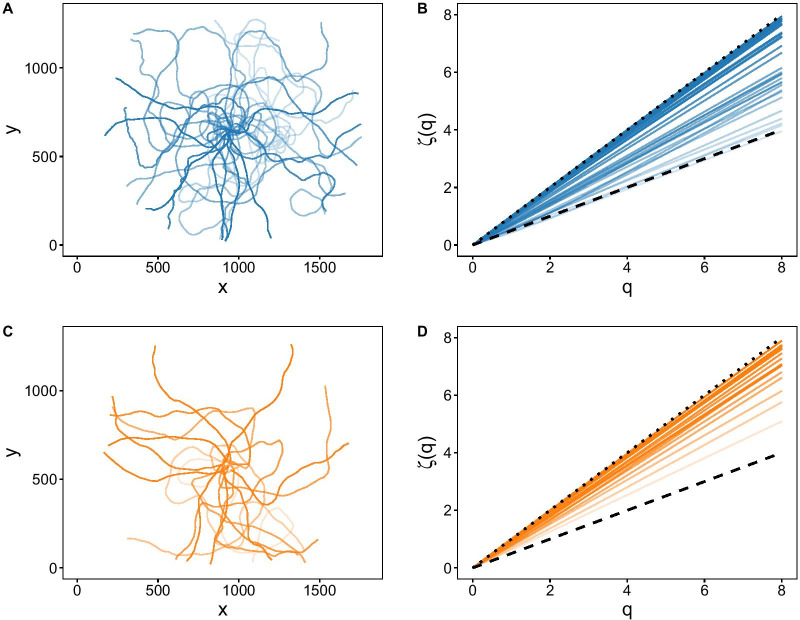
Analysis of empirical sea urchin trajectories. **a**, **c** Control (blue paths) and predator treatment (orange paths) sea urchin trajectories on an x, y coordinate system (n = 29 and n = 21, respectively). **b**, **d** Results from analysing control (blue lines) and predator treatment (orange lines) sea urchin trajectories using the qth order structure functions framework. Dotted and dashed lines in (**b**, **d**) correspond to the theoretical outputs of a ballistic (scaling exponents ζ(q) = q) and a Brownian trajectory (scaling exponents ζ(q) = q/2), respectively. Line transparency has been scaled by slope coefficient—solid colours indicate higher slope coefficients and increasing transparency indicates lower slope coefficients. The units of X and Y axes in (**a**) and (**c**) are pixels

Trajectories from sea urchins subjected to predator cues were straighter and faster, on average, than those of controls. In effect, the average straightness index and speed of the predator treatment group increased by 28% and 34% respectively, compared to the control group (Table 1, Fig. 2a,b). The scaling exponents of the q^th^ order moments (ζ(q)) allowed us to carefully assess the diffusive properties of individual sea urchin trajectories (see Figs. 2c, 3b,d, and Additional file 3: Fig. S3, Additional file 4: S4). In Figs. 2c and 3b,d, the steepest slopes (highest slope coefficients) correspond to marginal ballistic trajectories (ballistic motion occurring at ζ(q) slope = 1, dotted line in Figs. 2c, and 3b,d), while the gentlest slopes correspond to Brownian motion (when ζ(q) slope = 0.5, dashed line in Figs. 2c, and 3b,d). Finally, slopes between the Brownian and ballistic realms, correspond to superdiffusive trajectories. The diffusive properties of sea urchin trajectories under control conditions (i.e., in a featureless arena) ranged widely, whereas in the presence of predator cues, the range of sea urchin spreading behaviour was drastically limited (note the different shape of the violin plots in Fig. 2c and compare Fig. 3b with Fig. 3d). Trajectories from control urchins ranged from normal diffusion (Brownian motion) to marginal ballistic trajectories (Figs. 2c and 3a,b); while most of the sea urchins from the predators treatment displayed strongly superdiffusive or nearly straight-lined motion (marginal ballistic motion, Figs. 2c and 3c,d). Such differences in the variability of sea urchin spreading behaviour between treatments, were further highlighted by the fact that the best selected model evaluating the spreading behaviour of sea urchin trajectories as a function of treatment, required a specific variance structure to account for heteroscedasticity (i.e., different variance) among treatment levels (see methods, Fig. 2c).

**Table 1:** Summary of the linear models performed to test whether the dependent variables ‘mean speed’, ‘straightness index’, ‘scaling exponents ζ(q)’ and ‘directional entropy’ of sea urchin trajectories were different for the control (n = 29) and predator (n = 21) experiments. df, degrees of freedom

Response variable	Model type	Effect	df	Statistic	*P*
Straightness index (I_s_)	Linear model	Treatment	1	F = 4.66	0.036 *
		Residuals	48		
Mean speed	Linear model	Treatment	1	F = 11.35	0.001 **
		Residuals	48		
Slope of ζ(q)	General Least Squares	Treatment	1	χ^2^ = 9.48	0.002**
Directional entropy	Linear Model	Treatment	1	F = 7.02	0.011*
		Slope of ζ(q)	1	F = 38.52	0.000***
		Treatment*Slope ζ(q)	1	F = 2.03	0.161
		Residuals	46		

Significance codes: 0 ‘***’ 0.001 ‘**’ 0.01 ‘*’ 0.05 ‘.’ 0.1 ‘ ’ 1

**Fig. 4:**
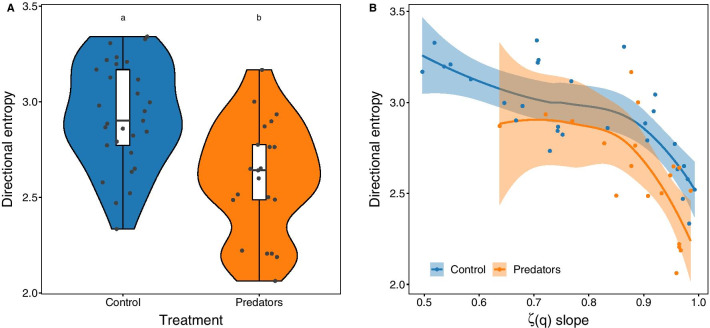
Directional entropy of sea urchin trajectories. **a** The directional entropy of sea urchins in the control treatment (blue, n = 29) was higher than for sea urchins subjected to predator cues (orange, n = 21). **b** The directional entropy of sea urchins’ trajectories decreased with steeper slopes of the scaling exponents of the *q*th order moments (ζ(q)), indicating that the predictability of individual trajectories increases as we move from Brownian motion towards ballistic movements. Points correspond to each individual observation and shaded areas in (**b**) correspond to 95% confidence intervals

In addition to being less variable in terms of diffusive properties, trajectories from sea urchins exposed to predator cues were also more predictable: the directional entropy of individual sea urchin trajectories from the predator treatment decreased by 13%, on average, compared to controls (Fig. 4a, Table 1). Logically, the directional entropy of sea urchin trajectories decreased with steeper slopes of the scaling exponents’ function (ζ(q)), meaning that as sea urchin trajectories varied from Brownian motion towards marginal ballistic, the predictability of their movement patterns increased (Fig. 4b, Table 1). Predictability appeared to increase faster from slope coefficients ranging from 0.8 to 1 (from superdiffusive to ballistic) than from coefficients ranging from 0.5 to 0.8 (Brownian to superdiffusive) (Fig. 4b).

## Discussion

A priori, few would expect the movement behaviour of sea urchins to be intrinsically complex, and rapidly adjustable to available contextual information. Our results, however, suggest the opposite: *Paracentrotus lividus* sea urchins showed complex movement behaviour in information-limited environments (our control trials), with a wide range of variability between individuals, while this variability was constrained as soon as information became available (predator chemical cues). Despite their old evolutionary origin (*Echinoidea* first appeared in the Ordovician) [19, 20], lack of cephalization [21], and homogenous external appearance, the trajectories that sea urchins displayed in information-limited environments ranged from Brownian motion to superdiffusion, and even marginal ballistic motion. Such variable behavioural repertoire appeared to be intrinsic to the species and emerged when the animals were in an information-limited (unconstrained) environment (control trials). Indeed, signatures of superdiffusive motion have been described for Late Cretaceous-Eocene trace fossils attributed to the echinoid ichnospecies *Scolicia* [52]. An ancient origin of such complex movement properties might suggest it is an intrinsic optimal behaviour for a broad range of heterogeneous landscapes (e.g. [53–56]). In contrast, as soon as some information became available (i.e., predator cues), inter-individual variability dropped, and trajectories became more homogeneous and predictable at the edge of ballistic motion. While the study of escaping strategies has progressed, until now most studies have focused on the initial phases of escape [14], but not on extended periods of time nor on analysing long individual trajectories (in the case of our trials, duration ranged from 8 to 104 min, see Additional file 4: Fig. S4, Additional file 5: S5). Our full-scale trajectory analysis with and without background predator chemical cues, allows for a more comprehensive understanding of the escape response of sea urchins.

One of the most unexpected results from this study has been the realization that despite their homogeneous morphology—we deliberately controlled sea urchin size—sea urchin behaviour was very variable in an information-limited (unconstrained) environment (control conditions). It was unexpected, indeed, because behavioural variation is often associated with morphological [57] or environmental variation [58, 59], something that our control trials were minimizing. Sea urchin diffusive patterns (Fig. 3a,b) were particularly variable within the control group. In the absence of environmental cues, the observed variability suggests the existence of complex intrinsic behaviour, including anomalous diffusion. The extent to which the observed variability arose from individuality is difficult to gauge by the fact that our experimental approach focused on comparing a population of control vs. conditioned sea urchins, instead of focusing on the individuals per se (i.e. trialling each urchin several times). Future studies should focus on individuals to effectively address the potential role of individual behaviour on population dynamics. Our experiment was neither designed to assess if such inter-individual behavioural differences were consistent over time or across contexts, and hence if they can be considered different ‘animal personalities’ [58, 59]. Nevertheless, the mere existence of such behavioural variation is the foundation on which natural selection can operate [60], potentially leading to adaptative solutions (as long as behavioural diversity implies genotypic diversity). A surge of recent research points to the importance of intraspecific variation for ecological and evolutionary processes [58, 61]. While traditionally most studies had focused on genetic variation concerning physiological, morphological, or other traits closely related to performance; the consequences of individual variation on behaviour have started to receive more attention in the last decade, becoming a hot topic [58, 62]. This is not surprising given that behaviour is a key factor mediating the interactions of individuals with their environment [63] and among themselves. In fact, individual behavioural variation has been shown to allow populations to cope with a broader range of environmental conditions, even influencing population stability and persistence [58, 59].

Conversely, sea urchins exposed to predator cues narrowed their range of spreading behaviours around ballistic-like movement patterns and low directional entropy, hence displaying more predictable escape trajectories. This contrasts to the avoidance reactions of many other prey organisms that tend to display more unpredictable behaviour when escaping from predators [5, 14, 16, 46, 64]. Such discrepancies might be explained by differences in predator foraging modes or strategies, which can determine the success of prey escape responses [10, 46]. For example, maintaining high velocities can be useful for prey that are hunted by predators using a simple pursuit strategy [64]. On the other hand, predation based on ballistic interception [64] requires the prediction of prey movement to plan a predator attack. Therefore, increasing the unpredictability of prey trajectories likely increases the chances of evading a predator’s ballistic interception [46]. In our case, since the predator cues to which urchins were subjected came from a slow-moving gastropod *(H. trunculus*) that hunts in a simple pursuit strategy [65, 66], an increase in urchin path unpredictability might not confer a selective advantage. In contrast, an increase in speed and straightness, leading to an almost ballistic spreading, might be the most advantageous strategy for outrunning a gastropod; hence the low unpredictability and overall convergence of most individuals tested to this escape strategy. Straight-line motion responses have been observed in other sea urchins escaping from sea stars (also pursuit hunters), for example, and may be a survival-enhancing response [67]. Ballistic trajectories maximise the distance an organism can travel in a given amount of time [15]. Similar escaping trajectories have been reported for pelagic plankton species (e.g. dinoflagellates [68] and copepods [69]). It has also been previously reported for molluscs, crustaceans, echinoderms, amphibians, reptiles, and mammals [3, 70]. The same strategy would not be appropriate if the predator was a faster-moving fish, and further empirical work should explore the kind of strategy that sea urchins would take under such circumstances. Model simulations show that the relative speeds of predator and prey are key to determine the most efficient predator foraging mode [13]. The same should be true looking at the predator–prey interaction from the viewpoint of the prey: prey antipredator behaviour is likely to change as a result of the relative speeds of predator and prey. In fact, sea urchins typically avoid fast predators (e.g., fish) altogether by hiding from them in crevices during daylight and foraging mostly at night. Such an avoidance strategy would not work for pursuit predators that can penetrate the refuges [10], such as the gastropods from our experiments.

*Paracentrotus lividus’* most remarkable functional trait might be its appetite for algal fronds and seagrass shoots, making it a key herbivorous species in Mediterranean seagrass meadows and algal communities [35, 71]. As a result of its functional importance, its movement ecology has been a matter of interest since the eighties, when Dance’s [72] meticulous observations showed that this species’ activity peaked at night, and that most animals made short-scale displacements. Other studies would later show that *P. lividus’* locomotor performance scaled with sea urchin size, that light could induce a negative phototactic response on this species [36], that their home range and displacement activity were different between protected areas and adjacent unprotected sites [73], and that movement behaviour differed between wild and captively-bred animals [74]. Our results add complexity layers to the suite of the potentially adaptive behavioural traits of this species. While freely moving (with no fear), overall movement covers broadly the superdiffusive regime which may be optimal to explore large-scale heterogeneous (patchy) and sparsely distributed resources [75, 76], such as the algae growing on the rocky reefs where these animals forage. In macroinvertebrates with limited visual abilities, chemical cues are one of the most important sources of information, and here we show, for the first time for this species, that predator chemical cues can induce strong changes in the species fundamental behavioural repertoire and variability. Given our model organism is considered a key herbivore in Mediterranean rocky reefs, it is reasonable to think that predator-induced behavioural changes may contribute to the observed differences in sea urchin control of macrophyte communities in protected vs. unprotected areas in the Mediterranean (resulting in so-called behaviourally-mediated trophic cascades, see for example [30]). Behaviourally-mediated trophic cascades have been widely reported when prey-species modulate their behaviour as a consequence of predator presence—the so-called non-consumptive effects of predators on prey (e.g. [77]). It was reasonable to expect a response from sea urchins subjected to very high concentrations of predator cues, when placed in an arena without acclimation. Further field research should determine the capacity of this species to also adapt their movement behaviour in situ. We have some hints that this is indeed possible, even if the concentration of predator chemical cues in the field are likely lower than those experienced by our laboratory specimens. In situ observations show that *P. lividus* increase the time allocated to sheltering in response to the presence of predatory fish [73]. Similarly, *P. lividus* inside marine protected areas (with high predator pressure) reduce their grazing activity in response to higher predation risk [30].

## Conclusions

The finding of intrinsically complex movement patterns in sea urchins, particularly in information-limited environments, and the instantaneous malleability of this behaviour in response to chemical cues is intriguing as echinoids lack cephalization and complex sensory organs [21], and have a very old evolutionary origin dating back from the Ordovician (> 400 Ma) [19, 20]. Our results highlight that fear of predators is an important driver of sea urchin movement patterns. While some movement patterns have been evolutionarily selected for optimising the search of food, other movement patterns might have been selected as optimal for escaping predators. In this context, the variability observed in sea urchin escape trajectories in a featureless environment (our control group) might be the foundation on which natural selection works to lead to survival-enhancing escaping strategies. All in all, the observation of anomalous diffusion, individual variability and the behavioural shift induced by predator cues, further highlight that the functional forms currently used in classical predator–prey models, which assume that both predator and prey behave as molecules in an ideal gas (Brownian motion) are far from realistic [8], with obvious influences on predator–prey population dynamics [18] and species coexistence [11]. Future modelling work should aim at incorporating the observed complexity in prey movement patterns, to make current predator–prey models more realistic.

## Supplementary Information

**Additional file 1**.** Fig. S1**. Downwelling irradiance measured at different sectors of the experimental arena’s floor. Light was measured using a HOBO device (Amplified Quantum Sensor, model SQ-200, Onset Computer Corporation, USA). Note that we found some heterogeneity in downwelling irradiance as shown by the different lower-case letters above each box, which correspond to significant differences according to Tukey HSD post-hoc tests.

**Additional file 2**.** Fig. S2**. Diagram showing the method used to calculate the initial (α_i_) and final (α_f_) heading angles. Note that we allowed five time steps before calculating the initial angle, to allow the sea urchin to settle after being moved from the holding tank.

**Additional file 3**.** Fig. S3**. Two examples (A, C, E, and B, D, F, respectively) illustrating the steps required to estimate the exponents ζ(q), from the slope of the linear trend of ∥∆Xτ∥ vs.* τ* in log–log plots. See methods for details. (A) and (B) correspond to the raw trajectories of two different sea urchins; (C) and (D) correspond to the ∥∆Xτ∥ vs. τ log–log plots, from where the exponents were estimated; and (E) and (F) are the final functions of scaling exponents ζ(q) plotted alongside the results of the ballistic motion (dotted line) and Brownian motion (dashed line). Shaded areas correspond to the 95% confidence intervals around the slope estimates from the linear regression. Note that on panels (C) and (D), τ’s (on the x-axis) are equally spaced as a result of using log-spaced τ’s when calculating sea urchin displacements, which then improves the compliance with the assumptions of linear regression.

**Additional file 4**.** Fig. S4**. Individual trajectories of the sea urchins from control trials. The number on the lower right corner of each panel corresponds to the total duration of the trajectory (since the frame rate we used was 30 seconds, the total number of steps for each trajectory can be calculated as Total_Duration*2). Line transparency has been scaled by slope coefficient—solid colours indicate higher slope coefficients and increasing transparency indicates lower slope coefficients. The units of X and Y axes are pixels.

**Additional file 5**.** Fig. S5**. Individual trajectories of the sea urchins from predator trials. The value on the lower right corner of each panel corresponds to the total duration of the trajectory (since the frame rate we used was 30 seconds, the total number of steps for each trajectory can be calculated as Total_Duration*2). Line transparency has been scaled by slope coefficient—solid colours indicate higher slope coefficients and increasing transparency indicates lower slope coefficients. The units of X and Y axes are pixels.

## Data Availability

All data and the R code for statistical analysis are available in the following GitHub repository [51].
