# Supplementary figures and images for: The scent of fear makes sea urchins go ballistic
Source: Mov Ecol. 2021 Oct 9;9:50. doi: 10.1186/s40462-021-00287-1 (PMC8502380; doi:10.1186/s40462-021-00287-1)

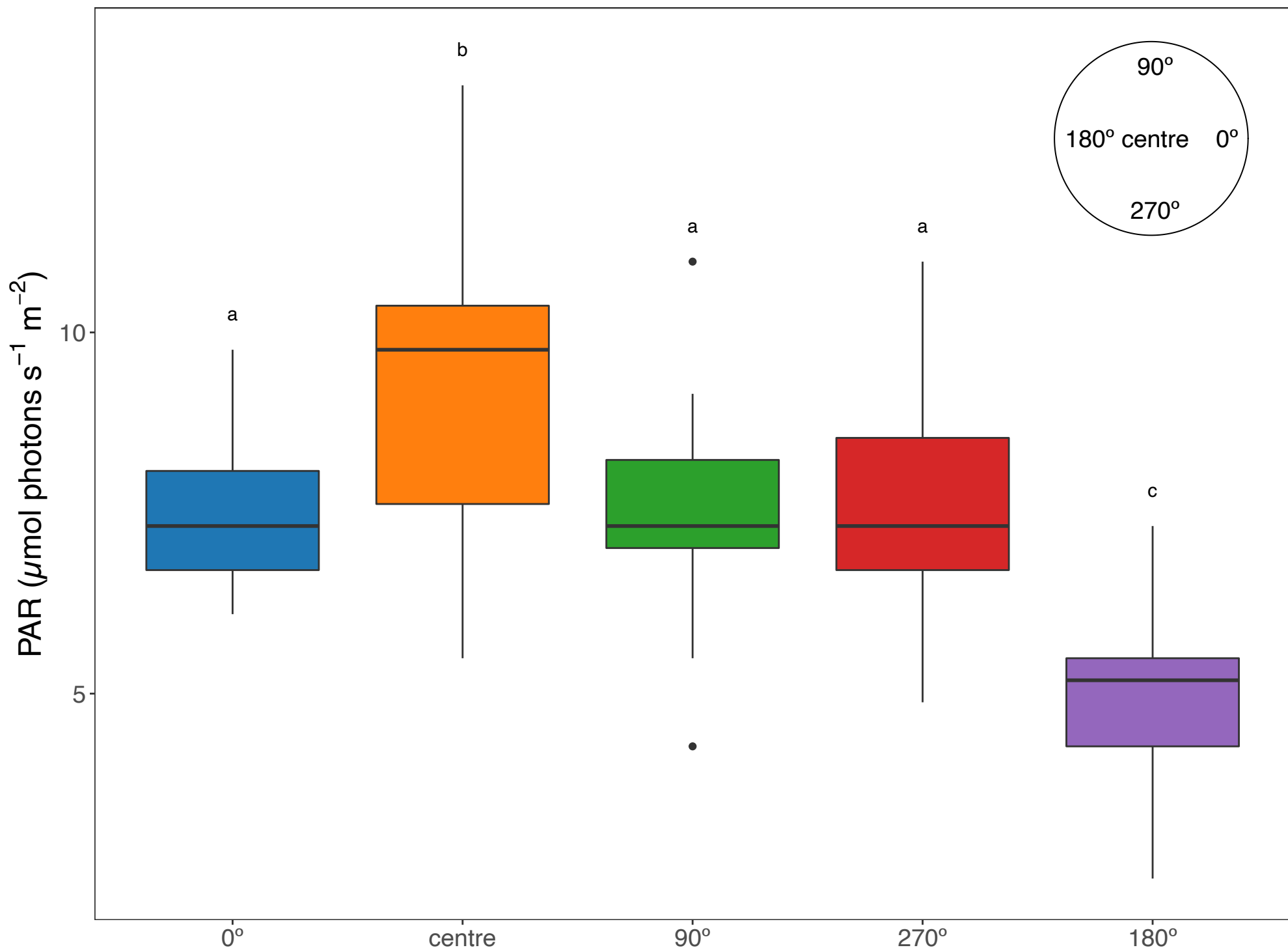

Supplement: Supplementary file 1 — Additional file 1. Fig. S1. Downwelling irradiance measured at different sectors of the experimental arena’s floor. Light was measured using a HOBO device (Amplified Quantum Sensor, model SQ-200, Onset Computer Corporation, USA). Note that we found some heterogeneity in downwelling irradiance as shown by the different lower-case letters above each box, which correspond to significant differences according to Tukey HSD post-hoc tests. [file 40462_2021_287_MOESM1_ESM.pdf]

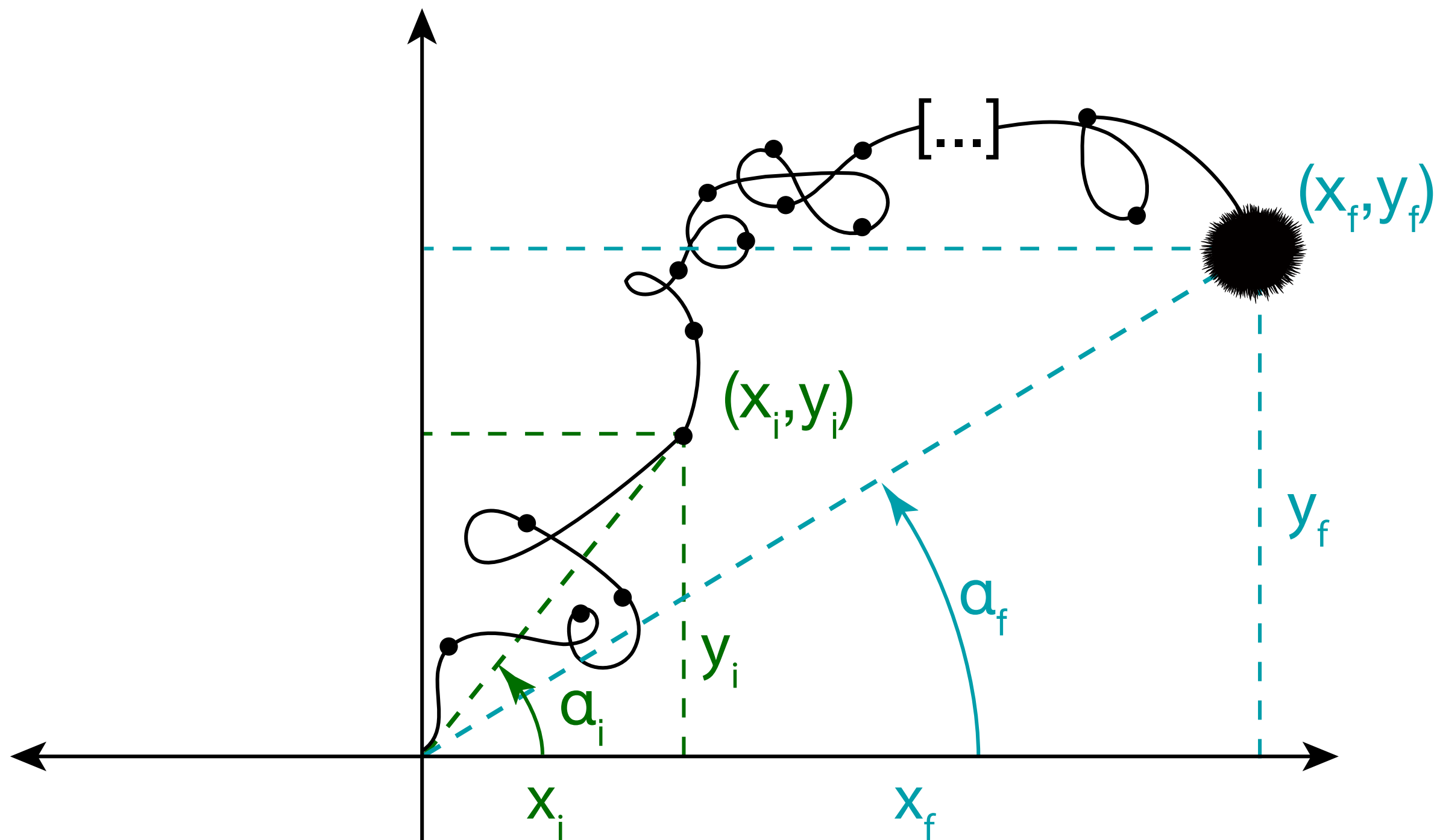

$$\alpha_i = \arctan(y_i, x_i)$$

$$\alpha_f = \arctan(y_f, x_f)$$

Supplement: Supplementary file 2 — Additional file 2. Fig. S2. Diagram showing the method used to calculate the initial (αi) and final (αf) heading angles. Note that we allowed five time steps before calculating the initial angle, to allow the sea urchin to settle after being moved from the holding tank. [file 40462_2021_287_MOESM2_ESM.pdf]

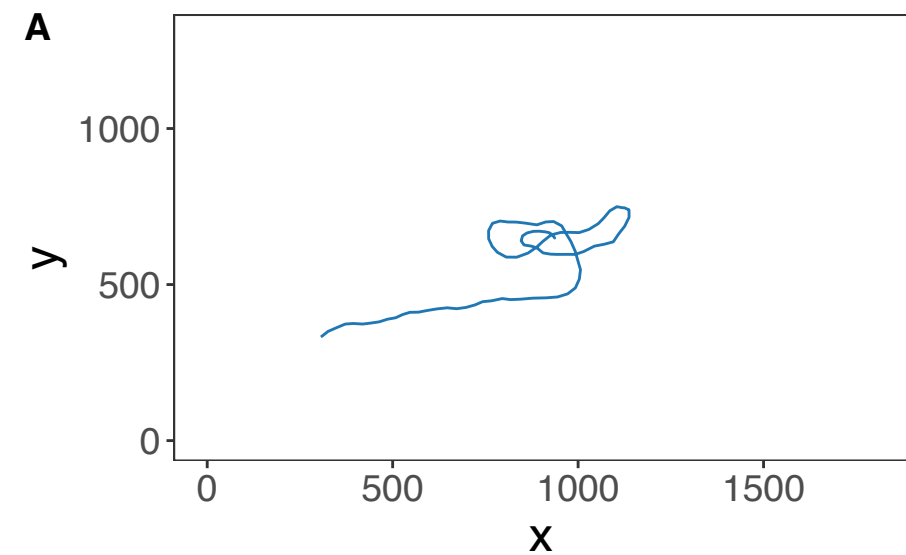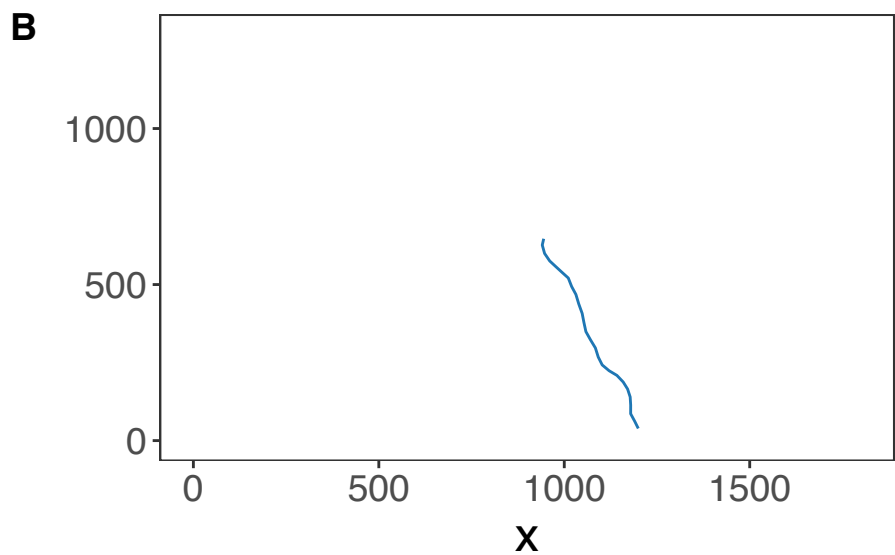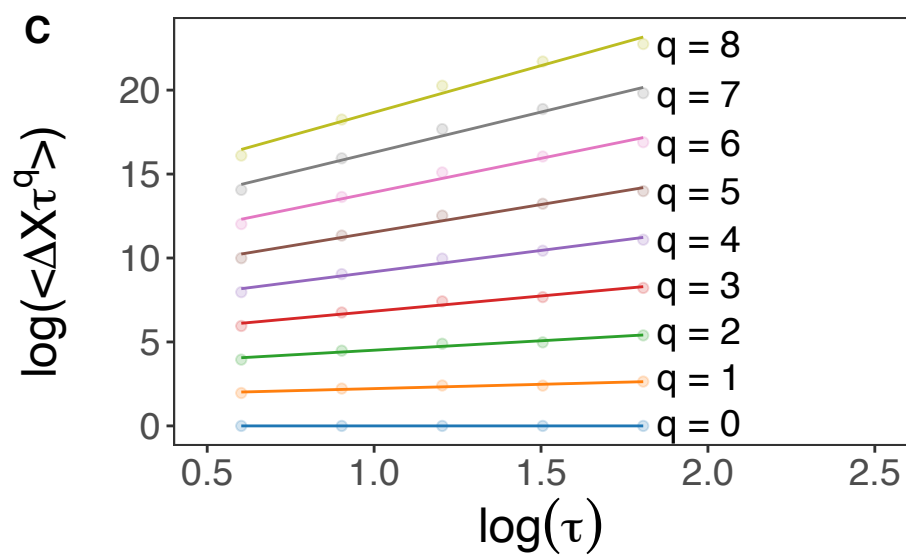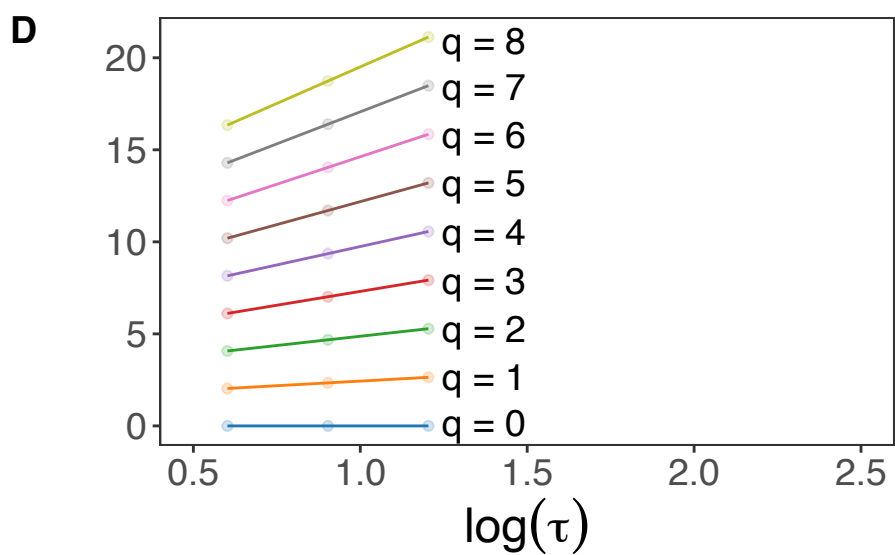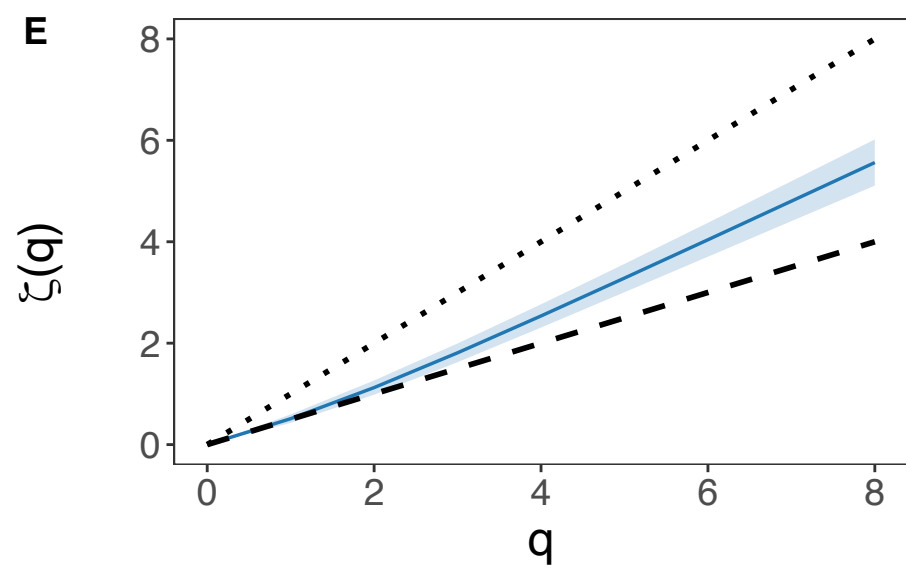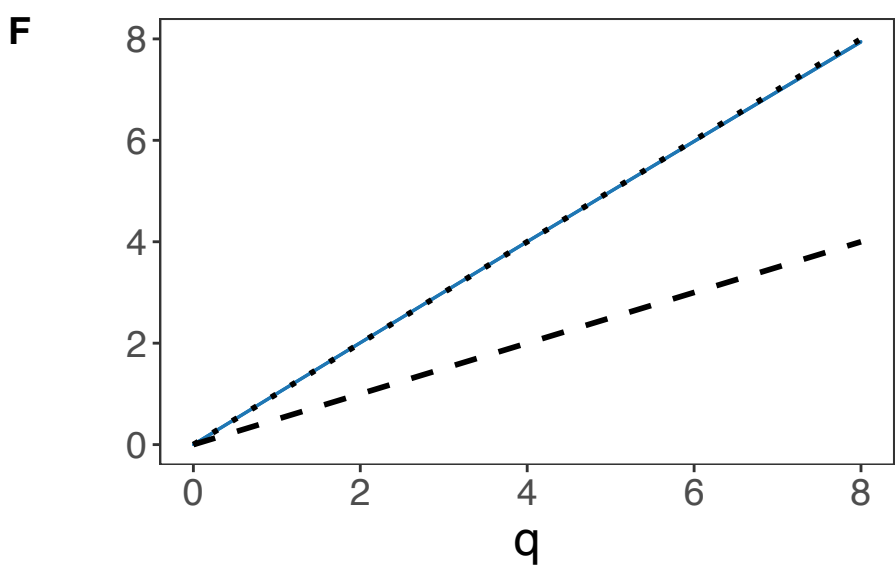

Supplement: Supplementary file 3 — Additional file 3. Fig. S3. Two examples (A, C, E, and B, D, F, respectively) illustrating the steps required to estimate the exponents ζ(q), from the slope of the linear trend of ∥∆Xτ∥ vs. τ in log–log plots. See methods for details. (A) and (B) correspond to the raw trajectories of two different sea urchins; (C) and (D) correspond to the ∥∆Xτ∥ vs. τ log–log plots, from where the exponents were estimated; and (E) and (F) are the final functions of scaling exponents ζ(q) plotted alongside the results of the ballistic motion (dotted line) and Brownian motion (dashed line). Shaded areas correspond to the 95% confidence intervals around the slope estimates from the linear regression. Note that on panels (C) and (D), τ’s (on the x-axis) are equally spaced as a result of using log-spaced τ’s when calculating sea urchin displacements, which then improves the compliance with the assumptions of linear regression. [file 40462_2021_287_MOESM3_ESM.pdf]

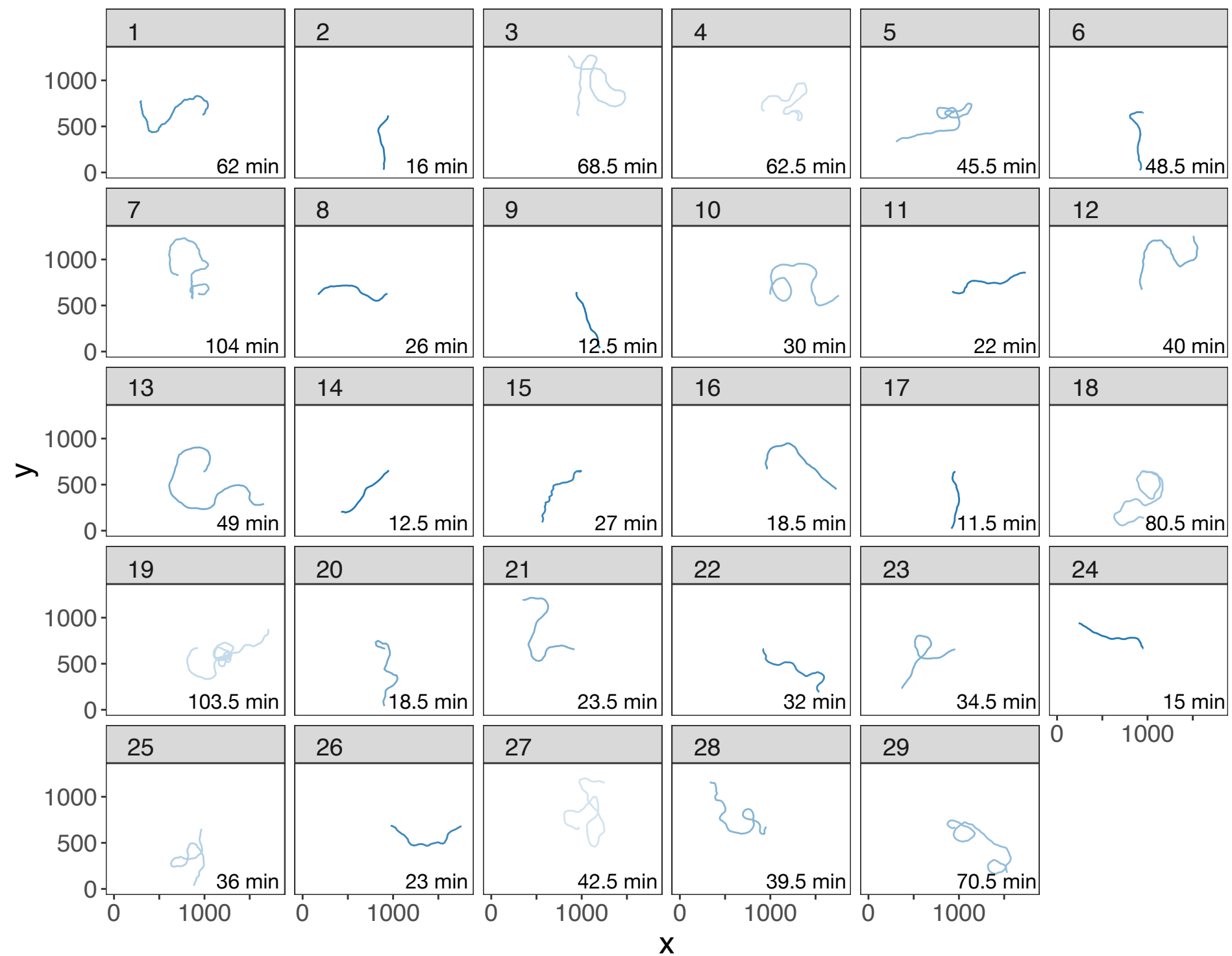

Supplement: Supplementary file 4 — Additional file 4. Fig. S4. Individual trajectories of the sea urchins from control trials. The number on the lower right corner of each panel corresponds to the total duration of the trajectory (since the frame rate we used was 30 seconds, the total number of steps for each trajectory can be calculated as Total_Duration*2). Line transparency has been scaled by slope coefficient—solid colours indicate higher slope coefficients and increasing transparency indicates lower slope coefficients. The units of X and Y axes are pixels. [file 40462_2021_287_MOESM4_ESM.pdf]

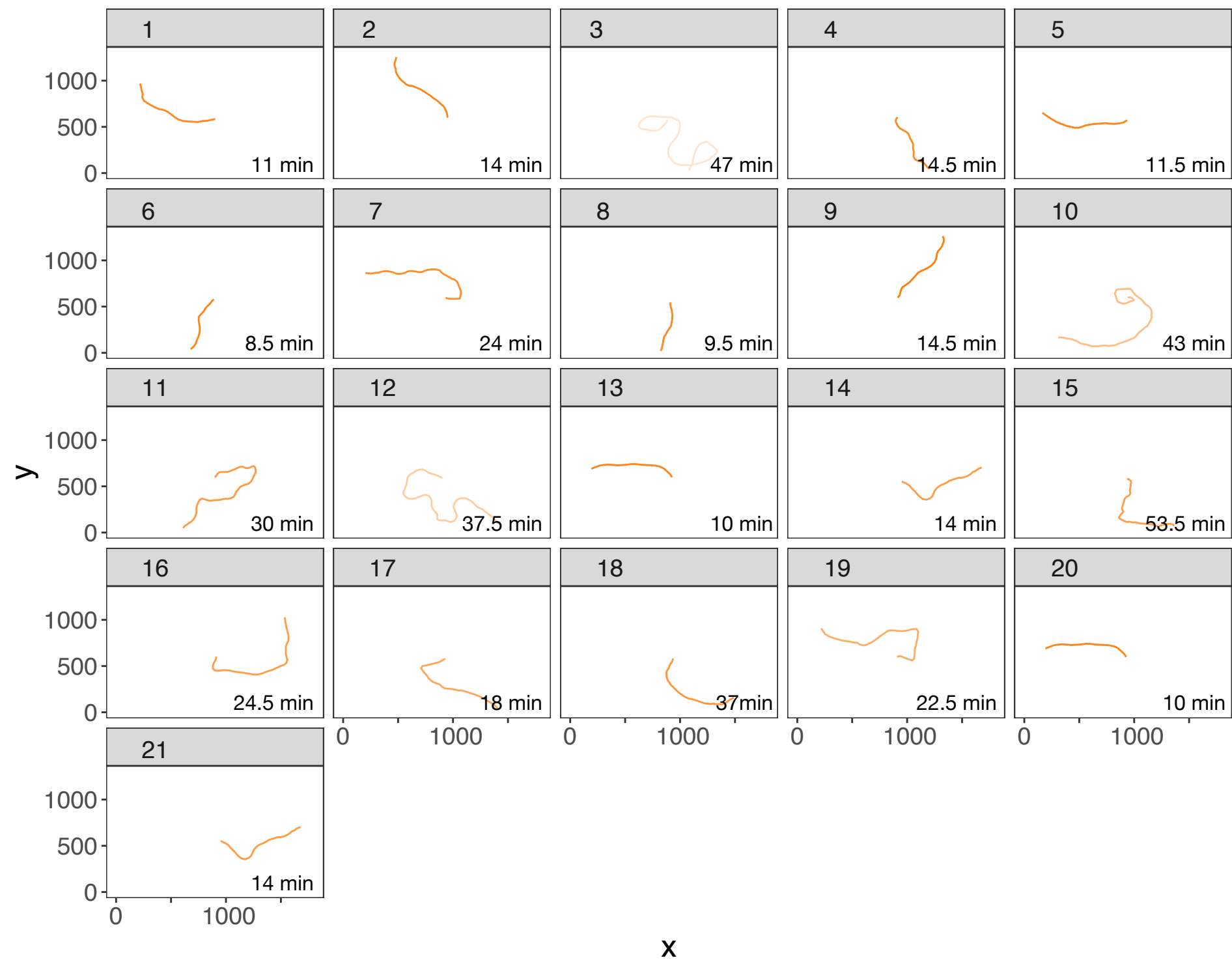

Supplement: Supplementary file 5 — Additional file 5. Fig. S5. Individual trajectories of the sea urchins from predator trials. The value on the lower right corner of each panel corresponds to the total duration of the trajectory (since the frame rate we used was 30 seconds, the total number of steps for each trajectory can be calculated as Total_Duration*2). Line transparency has been scaled by slope coefficient—solid colours indicate higher slope coefficients and increasing transparency indicates lower slope coefficients. The units of X and Y axes are pixels. [file 40462_2021_287_MOESM5_ESM.pdf]
